# Assessment of the response to cholera outbreaks in two districts in Ghana

**DOI:** 10.1186/s40249-016-0192-z

**Published:** 2016-11-02

**Authors:** Sally-Ann Ohene, Wisdom Klenyuie, Mark Sarpeh

**Affiliations:** 1 World Health Organization Country Office, 29 Volta Street Airport, Airport Residential Area, PO Box MB 142, Accra, Ghana; 2 School of Public Health, College of Health Science, University of Ghana, Accra, Ghana; 3 Komenda-Edina-Eguafo-Abirem Municipal Health Directorate, Central Region, Ghana

**Keywords:** Cholera, Outbreak response evaluation, Ghana

## Abstract

**Background:**

Despite recurring outbreaks of cholera in Ghana, very little has been reported on assessments of outbreak response activities undertaken in affected areas. This study assessed the response activities undertaken in two districts, Akatsi District in Volta Region and Komenda-Edina-Eguafo-Abirem (KEEA) Municipal in Central Region during the 2012 cholera epidemic in Ghana.

**Methods:**

We conducted a retrospective assessment of the events, strengths and weaknesses of the cholera outbreak response activities in the two districts making use of the WHO cholera evaluation tool. Information sources included surveillance and facility records, reports and interviews with relevant health personnel involved in the outbreak response from both district health directorates and health facilities. We collected data on age, sex, area of residence, date of reporting to health facility of cholera cases, district population data and information on the outbreak response activities and performed descriptive analyses of the outbreak data by person, time and place.

**Results:**

The cholera outbreak in Akatsi was explosive with a high attack rate (AR) of 374/100,000 and case fatality rate (CFR) of 1.2 % while that in KEEA was on a relatively smaller scale AR of 23/100,000 but with a high case fatality rate of 18.8 %. For both districts, we identified multiple strengths in the response to the outbreak including timely notification of the district health officials which triggered prompt investigation of the suspected outbreak facilitating confirmation of cholera and initiation of public health response activities. Others were coordination of the activities by multi-sectoral committees, instituting water, sanitation and hygiene measures and appropriate case management at health facilities. We also found areas that needed improvement in both districts including incomplete surveillance data, sub-optimal community based surveillance considering the late reporting and the deaths in the community and the inadequate community knowledge about cholera preventive measures.

**Conclusion:**

The assessment of the cholera outbreak response in the two districts highlighted strengths in the epidemic control activities. There was however need to strengthen preparedness especially in the area of improving community surveillance and awareness about cholera prevention and the importance of seeking prompt treatment in health facilities in the event of an outbreak.

**Electronic supplementary material:**

The online version of this article (doi:10.1186/s40249-016-0192-z) contains supplementary material, which is available to authorized users.

## Multilingual abstracts

Please see Additional file 1 for translations of the abstract into the five official working languages of the United Nations.

## Background

Cholera, an acute bacterial diarrhoeal disease, caused by the bacteria Vibrio cholera is usually transmitted through water or food contaminated with faecal matter [1]. An estimated 20 % of those who are infected develop acute watery diarrhea. The diarrhoea becomes very severe in about 10–20 % of these individuals and may be accompanied by vomiting. Without prompt and adequate treatment, these patients lose large amounts of fluid and salts leading to severe dehydration and death within hours. With appropriate treatment which largely hinges on fluid replacement, case fatality rate is less than 1 % [1].

Annually about 2.8 million cholera cases are reported globally and deaths from cholera are estimated to range from 28,000 to 142,000 [2]. Cholera has become endemic in Africa with large epidemics occurring in different parts of the continent [3, 4]. Over the course of 2007 to 2011, more than 100,000 cases of cholera were reported to WHO annually from at least 20 African countries including Ghana with case fatality rates reaching about 3 % [5]. Since 1970, when Ghana experienced the first cholera outbreak, the country has had many more epidemics occurring over the years [6–11].

Because of the potential for cholera to become a major public health problem and spread quickly locally and even internationally, it is important to have a well-coordinated, timely and effective response in the event of an outbreak. Following an outbreak it is recommended that an assessment of the outbreak response is undertaken to identify strengths and weaknesses to inform planning for improved preparedness and response towards future outbreaks [12]. Despite the recurring cholera outbreaks in Ghana, very little has been reported on assessments of outbreak response activities undertaken in affected areas. In 2012, an outbreak of cholera affected 53 districts in nine regions in Ghana with 9548 cases and 100 deaths and the case fatality rate (CFR) was 1.0 % nationally [10, 13]. Districts reporting cases were variably affected with attack rates and cases fatality rates ranging from 0.8 to 374 per 100,000 population and 0 to 18.8 % respectively [13]. In this paper we present the findings from the assessment of response activities undertaken in two districts, Akatsi District and Komenda-Edina-Eguafo-Abirem (KEEA) Municipal as they were the districts with the highest attack rate and case fatality rate respectively in the 2012 cholera epidemic in Ghana.

## Methods

### Study areas

**Fig. 1:**
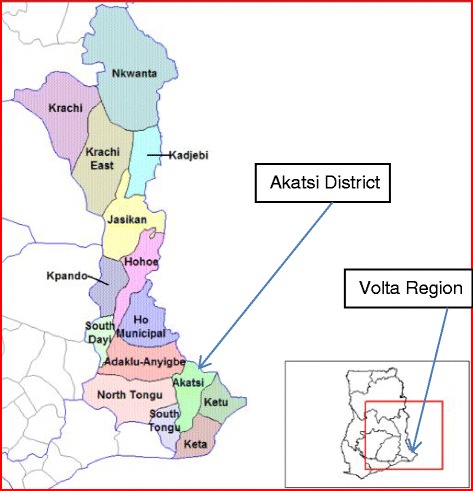
Map showing the location of Akatsi District in the Volta Region of Ghana (Source Wikipedia)

**Fig. 2:**
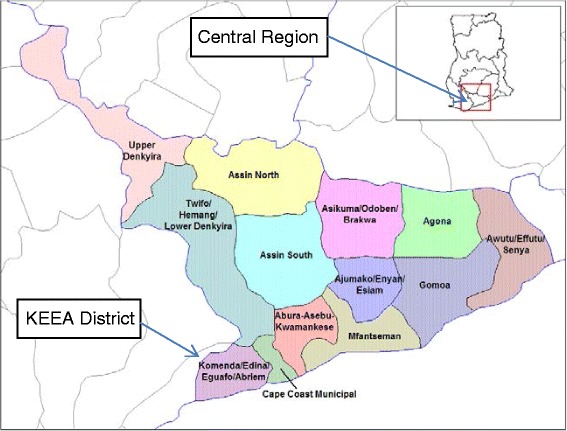
Map showing the location of KEEA District in the Central Region of Ghana (Source Wikipedia)

Akatsi District, shown in Fig. 1, is located in the south eastern part of the Volta Region of Ghana (latitude 60S 70N and longitudes 00W 10E) [14, 15]. Even though for administrative purposes Akatsi District has now been divided into Akatsi North and Akatsi South Districts, as at the time of the outbreak in 2012 however, it was one district. It is divided into five health sub-districts namely Akatsi, Avenorpeme, Gefia, Wute and Ave-Dakpa for administrative and management purposes. Komenda-Edina-Eguafo-Abirem (KEEA) Municipal in the Central Region of Ghana, shown in Fig. 2, covers an area of 452 km^2^and is located between longitude 1° 20′ West and 1° 40′ West and latitude 5° 05′ North and 5° 15North [16]. There are five sub-districts namely Kissi, Elmina, Komenda, Ankaful and Agona sub-districts each with a health center and Ankaful Hospital serves as the district hospital. With regard to previous cholera outbreaks, Akatsi District had not encountered any in over 5 years while KEEA experienced outbreaks in 2010 (2 cases, no death) and 2011 (44 cases, 1 deaths, CFR 2.3 %).

### Study design and data collection

We embarked on a retrospective assessment of the 2012 cholera outbreak response in the two study districts. We made use of the WHO cholera evaluation tool obtained from the WHO document, *Cholera outbreak: assessing the outbreak response and improving preparedness* [12]. To assess the successes and gaps, we inquired about the actions that were undertaken during the response and compared them to what was expected to be carried out in an outbreak situation as outlined in the document [12]. The tool provides a framework to assess the components of the response to a cholera outbreak with the view to highlight the strengths and weaknesses and make recommendations to enhance preparedness and improve the response to outbreaks that could occur in the future [12]. Among the areas the tool outlines for review of strengths and weaknesses are the organization of the response, outbreak detection, laboratory confirmation and surveillance activities, case management, control of the environment including water, sanitation and hygiene activities and measures to control the spread of cholera in the community. The components of the tool are summarized in Table 1. To obtain our data, we interviewed key members of the District Health Management Teams (DHMT) of both districts who were involved in the response to access data on those affected by the outbreak and the conduct of the response as well as information on previous cholera outbreaks in the preceding 5 years. The personnel interviewed included the district director of health service and the district disease control and surveillance officers. We also consulted the health staff who were involved in the management of cholera cases in the Akatsi and KEEA district hospitals and health centers where the cases were treated. We reviewed surveillance records and case definitions used to define cases, outbreak reports and health facility records and collected data on age, sex, area of residence, date of onset of illness and date reporting to health facility of cholera cases and district population data which we entered on a 2010 Microsoft Excel worksheet for analyses. We conducted descriptive analyses of the outbreak data by person, time and place. Utilizing the information on the outbreak response activities accessed from the various sources including the responses from the interviews, we tabulated the key strengths and weaknesses for each district under the respective components of the evaluation tool.

**Table 1:** Tool for evaluation of cholera response activities

Response areas	Components	Strengths	Weaknesses
Organization of the response	Organization of the response – cholera coordination committee – control measures – monitoring of the activities		
	Involvement of international partners – coordination of partners – project proposals		
	Information management – information dissemination – media involvement – coordination with media		
Surveillance and laboratory confirmation	Surveillance – data for action – outbreak investigation		
	Outbreak detection – quality of early warning system – quality of background information – flow of epidemiological information – first control measures taken		
	Outbreak confirmation – laboratory confirmation of diagnosis – case definition		
Case management	Case management: treatment – rehydration therapy – antibiotics – preventive measures (isolation, hygiene)		
	Reduction of mortality – case-fatality rate – training of health care workers – cholera treatment units in place		
	Hygiene measures in health care facilities – organization of cholera treatment units – disinfection – water supply		
Control of the environment	Safe water – measures to ensure safe drinking-water. – chlorination of water sources		
	Sanitation – access to sanitation facilities – hygiene		
	Funeral practices – official recommendations to communities, health care workers and funeral organizers		
Control of the spread in the community	Involvement of the community – education campaign (messages and channels) – active case-finding		
	Safe food – marketplaces – street food handlers		

Derived from *Cholera outbreak: assessing the outbreak response and improving preparedness*. Geneva: World Health Organization; 2004 [12]

The exercise was a review of public health practice and risk was considered minimal. Permission was sought from the Disease Surveillance Division of the Ghana Health Service and Central and Volta Regional Health Directorates and DHMT of the two districts. Informed verbal consent was obtained from those interviewed. There was no personal identifying information in the data collected and all data was handled with strict confidentiality.

## Results

### Events and epidemiology in the cholera outbreak

#### Akatsi District

**Fig. 3:**
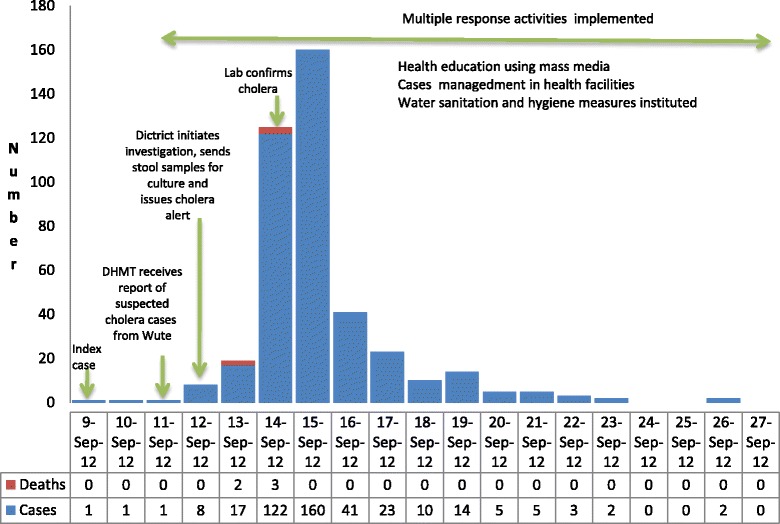
Epidemic curve of cholera cases and deaths by date reported, Akatsi District, 2012

**Fig. 4:**
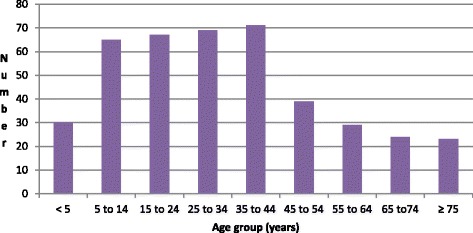
Age Distribution of 2012 Akatsi District cholera cases

We found that on 11th September 2012, the Medical Doctor at the District Hospital alerted the DHMT about 3 cases on admission with diarrhea and vomiting that were suspected to be cholera. The three were from the Wute Sub-district. Subsequently over the course of the epidemic which lasted up to 26th September when the last 2 cases was recorded, we noted that a total of 422 cases of cholera were reported. This was one case less than the 423 cases reported at the national level. The attack rate was 374/100,000 in the estimated population of 112,836. There were 5 deaths, CFR 1.2 %. We noted that all the five deaths occurred in the Wute sub-district (four occurred in the home and 1 died on arrival at the Wute Health Center). The district officials reported that upon investigation of the deaths, they found one of the affected individuals was a person who after successfully being treated for cholera at the district hospital was re-infected with cholera after drinking untreated contaminated water from River Tordzi, the community water source. They indicated this individual had expressed disbelief that the source of the cholera was from the water and it was of their opinion that this reflected inadequate knowledge about cholera transmission among some community members. We identified data for 418 of the cases for analyses and noted a few missing data for some of the variables. About two-thirds of cases, (285 in number), were recorded over 2 days 14^th^ and 15^th^ September. Figure 3 shows the epidemic curve for the district. The majority of cases, 305 (making up 73 %) came from Wute, while 90 (22 %) were from North Tongu, the district to the west of Akatsi and 21 (5 %) were from the Akatsi sub-ditrict. Out of the 387 for whom sex was indicated on the line list, 52 % were male. About 65 % of the people affected were between the ages of 5 and 44 years. The age distribution of affected cases is portrayed in Fig. 4. Ninety-eight cases, almost a quarter of the total, required admission.

#### Komenda-Edina-Eguafo-Abirem (KEEA) Municipal

**Fig. 5:**
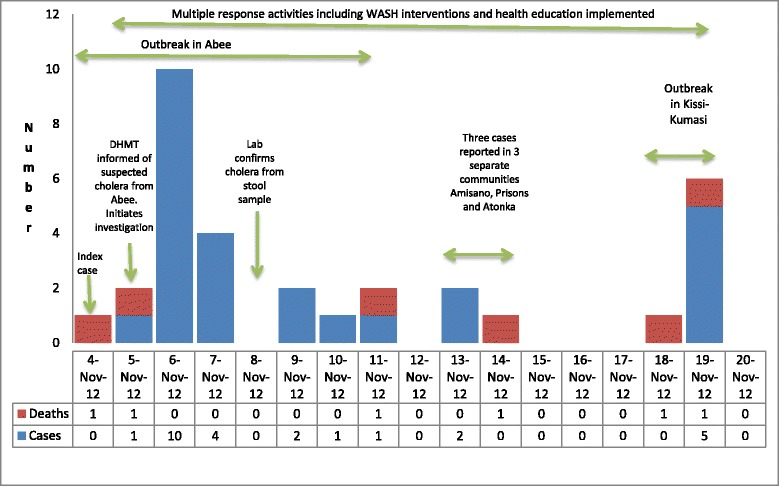
Epidemic curve of cholera cases and deaths by date reported, KEEA District, 2012

**Fig. 6:**
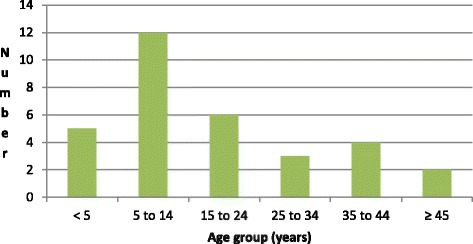
Age distribution of 2012 KEEA District cholera cases

In KEEA, we observed that the first case in the 2012 outbreak was reported on 4th November when the DHMT was informed of a death in a male adult, following symptoms of diarrhea and vomiting in the Abee Community. Subsequently 20 more people from the same Abee community developed similar symptoms most of whom reported to Ankaful Hospital, the district hospital. Three other communities Amisano, Prisons and Atonkwa reported a case of cholera each over 13th to 14^th^ November. Kissi-Kumasi community recorded 7 cases (22 %) over 18^th^ to 19^th^ November. No cases were reported after 19^th^ November 2012. The epicurve in KEEA is illustrated in Fig. 5. With an estimated population of 138,410 we calculated the attack rate to be 23/100,000. Out of the 32 cases in total, there were six deaths. The district attributed the deaths to delay in reporting to the health facility for management indicating that five of these deaths occurred in the community (3 in Abee, 1 in Kissi-Kumasi and 1 in Atonkwa). The sixth from Kissi-Kumasi was pronounced dead on arrival at the Cape Coast Hospital. We discovered on review of the Ankaful Hospital admission and discharge records that an elderly man died within hours of admission on 31^st^ October 2012 after complaints of diarrhea and vomiting. This case, which could have been the index case of cholera in the district, was not reported to the DHMT and so was not investigated. Almost three-quarters of the cases (72 %) were below 25 years with the 5 to 14 year group recording the highest number of cases 12 (38 %), as portrayed in Fig. 6. More than half of the cases, 17 in number (53 %), were males. Even though 32 cholera cases was the figure reported at the national level, we noted that the district records stated that there 38 cases of cholera in total during the 2012 outbreak.

### Outbreak detection and confirmation

#### Akatsi District

We found that the Akatsi DHMT was informed of the cholera outbreak within 48 h of the index case reporting and immediately launched an investigation on 12^th^ September. Stool samples were sent to the Volta Regional Hospital laboratory where Vibrio cholera O1 was confirmed by stool culture. We were informed that the outbreak investigation traced the source of the outbreak to River Tordzi the major source of drinking water for the Wute community. Cholera vibrio was isolated from water sampled from the river and was the same strain isolated from the stool sample. The River Tordzi runs through Adaklu-Anyigbe District and North Tongu District and downstream through Akatsi District. We gathered from the district health officials that they found out later that cholera had broken out upstream in North Tongu but the Akatsi DHMT was not informed by their counterparts.

#### Komenda-Edina-Eguafo-Abirem (KEEA) Municipal

We noted that within 24 h of occurrence, the DHMT was alerted of a death following diarrhea and vomiting in Abee Community and of cases reporting to Ankaful Hospital with similar symptoms and embarked on an investigation on 5^th^ November. Samples taken from patients on 6^th^ November were confirmed for Vibrio cholera O1 by stool culture 2 days later at the Central Regional Hospital in Cape Coast and Public Health and Reference Laboratory in Accra. The KEEA district health officials reported that risk factors for the outbreak in Abee included an overflowing pit latrine that was contaminating a nearby stream which served as drinking water to some community members while in Kissi-Kumasi, contamination was from the public toilet close to the house of affected cases. They also cited inadequate potable water and open defecation in the communities as contributory factors.

### Assessment of the outbreak response

We identified various strengths and weaknesses in the response activities in the two districts and these are highlighted in Tables 2 and 3. For both districts, we found similarities in the strengths that were identified in the outbreak response. For example response activities were coordinated by multi-sectoral committees in the two districts. Similarly in both, all cases managed at the health facilities survived pointing to appropriate case management at health facilities. The timely reporting of suspected cholera cases to the DHMT in both districts triggered prompt confirmation of cholera and activated response activities. Educational campaigns on cholera preventive measures including hand washing with soap, making water safe before drinking and appropriate waste disposal were undertaken in the affected communities in both districts following the outbreak. Concerted efforts were taken to disinfect water sources and other possibly contaminated community sites and facilities including public toilets, pit latrines and waste disposal sites. We also found some similarities in the areas that needed improvement in both districts. These included incomplete and inadequate analyses of surveillance data, discrepant figures of total cases reported at national level and the sub-optimal community based surveillance considering the late reporting and the deaths in the community, inadequate community knowledge about cholera and limited availability of management materials and protocols in the health facilities. On the other hand there were some contrasting aspects between the districts in some components. Whereas Akatsi District lacked an epidemic emergency plan, KEEA had one. KEEA conducted training for their health staff in case management while this was not done in Akatsi (Table 3).

**Table 2:** Summary of evaluation of cholera outbreak in Akatsi

	Activities and strengths	Weaknesses/Areas for improvement
Organization of the response	• Multi-sectoral Emergency Preparedness committee activated and divided into 5 teams with assigned roles. Members included DHMT, District Hospital Medical Superintendent, District Assembly, pharmacist, District Chief Executive, District Environmental Officer, Ministry of Food and Agriculture (MOFA) director, security forces, education director, Member of Parliament) • Daily meetings were held to review activities and re-assign roles • There was documentation of activities conducted including outbreak investigation, interim and end of outbreak reports.	• There was no epidemic preparedness plan • Available reports lacked adequate analyses of affected persons in time, person and place and spot map
Surveillance and laboratory confirmation	• The DHMT was rapidly notified by hospital staff when initial cases reported • The line list of cases was compiled at facilities, updated at close of day and data transmitted to regional level daily by call and to lower levels to keep them on the alert • There was availability of sample transport medium which facilitated early laboratory confirmation • Water samples from 5 communities were tested with drug sensitivity testing conducted for isolated organisms	• Considering late reporting and deaths in community, the performance of the community-based surveillance system was sub-optimal • Poor communication channels with North Tongu DHMT so that the news of the cholera outbreak was not transmitted
Case management	• Cholera treatment centers were set up away from other operations of the health facilities • No deaths from Cholera occurred in the health facilities • Outreach to support case management in Wute Health Center (HC) was undertaken by doctor from the District Hospital • Infection prevention and control measures were ensured in district hospital with adequate water, proper disposal of waste and disinfection of linen & clothes • Logistics were available and replenished when stocks became low • Private facilities participated in case management • Cases from hard to reach areas were transported to health facilities for treatment	• No case definition, assessment protocols nor management flow charts were made available to health workers • No refresher training of staff in case management • Waste disposal facilities at Wute HC was inadequate
Control of the environment	• Water sources in affected communities and public toilets were chlorinated • Faulty boreholes were repaired • Dead bodies and their homes were fumigated before supervised burial by environmental health officers • Dead bodies were not released to families for funerals but buried under supervision of environmental health officers	• There was no coordination with North Tongu District in environmental disinfection
Control of the spread in the community	• Education on food safety, hand washing, waste disposal in schools, communities and markets was undertaken • There were regular radio health education and mobile van announcements in the communities • Food stuff from affected communities were barred from the market • Faulty boreholes repaired and toilet facility built in communities lacking them and people were informed to use them instead of open defecation. • Prophylaxis given to contacts of cases • Restrictions on the movement of animals was enforced with strays being confiscated	• Inadequate community sensitization regarding contaminated drinking water as the source of cholera. One death was attributed to re-infection from contaminated water

**Table 3:** Summary of evaluation of cholera outbreak response in KEEA

	Activities and strengths	Weaknesses/Areas for improvement
Organization of the response	• Multi-sectoral Cholera task force involving the Municipal Chief Executive, the District Health Management Team, the District Assembly, District Environmental Officer (EO), Ghana Education Service (GES), National Commission for Civic Education, media, fire service, police and National Disaster Management Organization was activated. • A district Cholera emergency plan was developed and implemented. • Health education with media collaboration including radio discussions and community FM announcements was undertaken. • Field investigations conducted in Abee and Kissi-Kumasi were documented in a written report. • Central Region Health Administration paid supportive visits and supplied logistics for the response. • Rapid response to contain outbreak	• No end of epidemic report with analysis of cases by time, person and place nor evaluation with recommendations to guide future preparedness and planning activities
Surveillance and laboratory confirmation	• Rapid notification of DHMT by hospital staff when initial cases reported (within 24 h) • Carrie Blair media were available for sample transport • Samples were transported to laboratory for confirmation of cholera in timely manner • The facility registers were reviewed daily for new cases to facilitate community follow up. • Cholera data was transmitted to the Regional level and also shared with the sub-districts • A line list of cases was compiled	• Hospital staff were not informed about lab results when cholera was confirmed • Discrepancy in number of cases reported to national level and number on line list • Late reporting of deaths in community level suggests gaps in community-based surveillance
Case management	• Cholera treatment center set up for isolation of cases in Ankaful Hospital • Good management: no deaths in the hospital during outbreak • Staff trained in management • Infection prevention and control measures observed: adequate water ensured, proper disposal of waste and disinfection of linen & clothes • Supplies were available and replenished when stocks became low • Emergency stock of supplies available in hospital at the time of assessment	• No case definition, assessment protocols nor management flow charts were made available to health workers • A patient admitted on 31 Oct 2012 with diarrhea and vomiting and died same day was probably a suspected cholera case that was missed.
Control of the environment	• Water sources and public toilets were disinfected with chlorine • The overflowing pit latrine in Abee was emptied following outbreak • Dead bodies were fumigated before supervised burial by environmental health officers	• The overflowing latrine had been reported to the District Assembly earlier but no action had been taken before the cholera outbreak
Control of the spread in the community	• Education on food safety, hand washing, waste disposal was undertaken in schools, communities and markets • Byelaws against open defecation were instituted • Prophylaxis given to contacts of cases • A community survey was conducted to assess the effectiveness of the health education • Community volunteers were mobilized to participate and support in education • The Hospital Public Health Unit (PHU) participated in social mobilization campaign in the communities	• Practically all the deaths occurred in the community suggesting inadequate community knowledge about: o early initiation of oral fluid replacement on onset of symptoms; and o early reporting to health facilities

## Discussion

This paper highlights the events of cholera outbreaks that occurred in 2 districts in Ghana Akatsi and KEEA and the review of the response activities. For both, probable exposure to the contaminated community source of drinking water may have been responsible for the outbreak. The outbreak in Akatsi was explosive with a high attack rate and was traced to a contaminated stream which served as common source of infection. That in KEEA was on a relatively smaller scale with a high case fatality rate attributed to late reporting of cases to the health facility. Virtually all deaths recorded in the 2 outbreaks occurred outside the health facility. Following implementation of response measures including massive community education and water, sanitation and hygiene (WASH) activities, the outbreaks were controlled in 2 weeks.

Cholera remains a threat to areas where access to safe drinking water and adequate sanitation cannot be assured [8]. Considering that 35 % of households in Ghana obtain drinking water from a source that is not considered protected or safe and only 15 % have access to improved sanitation, the risk of cholera outbreaks remains high for several communities in Ghana [17]. Therefore in tandem with long term steps to improve WASH facilities, having an enhanced surveillance system in place, high community awareness about preventive measures and a preparedness plan for mounting a timely and appropriate response are imperative preparedness activities for effective prevention and control of cholera outbreaks [5, 8, 12]. Ghana is implementing the World Health Organization Regional Office for Africa (WHO-AFRO) Integrated Disease Surveillance Response (IDSR) approach for public health surveillance which uses standard case definitions to diagnose and track priority diseases for public health action [18]. Cholera is listed as one of the epidemic prone diseases and a single suspected case requires immediate notification within 24 h of occurrence to the next level of the health surveillance system along the chain from the community to health facility, sub-district, district, region and national levels. The district, the decentralized administrative unit in the country, has the responsibility of leading investigations into suspected outbreaks within 48 h of notification. The objective is to generate information making use of surveillance and laboratory data to guide relevant and timely public health response. As part of preparedness, it is also expected that the district sets up a multidisciplinary district public health management committee which develops contingency plans with the objective of strengthening the capacity of the district to respond to outbreaks or public health events. Following the response to the event, the district staff are expected to document actions taken and prepare reports highlighting these activities, the outcomes and recommended public health actions to improve preparedness. In the use of the WHO evaluation tool, the two districts had a number of strengths in common such as the multi-disciplinary approach to coordinating response activities, the timely investigation by the DHMT within the recommended 48 h of the prompt notification of the suspected outbreak with the resultant public health actions initiated to control the outbreaks [18]. The surveillance system could however had been more pre-emptive if the community based surveillance (CBS) component had been more robust in both districts and in the case of Akatsi Distict, the lines of communication with the neighbouring district which had an ongoing cholera outbreak had been more open as recommended [18, 19]. Strengthening CBS involves creating awareness about diseases of public health importance using simplified case definitions and tools so that community participation in the monitoring, detection, reporting and response to events of public health importance in the community can be enhanced. Subsequently, the strong CBS system can serve as an important alert or warning mechanism in a pre-epidemic period [18, 19]. The various multi-sectoral response activities mounted by the two districts including extensive health education avenues some of which involved using community volunteers to impart information on preventive measures, appropriate case management in the health facilities, decontamination of water sources and improvement of waste disposal facilities facilitated the control of the outbreak. It is commendable that the health workers at the attending facilities were up to the task and managed the cases appropriately. Even though in both districts, the case fatality rate was above the WHO acceptable rate of 1 % this was not a reflection of poor case management in the health facilities as all cases reporting to the facilities survived [12]. Nevertheless, in consideration of possible staff turnover and new staff coming in, it is useful to have available treatment protocols and management flow charts to assist staff in assessment and management and to ensure that they are trained in their use [12]. The deaths in the community especially in KEEA were related to delayed care-seeking. Consequently, the district health officials also need to explore the issues of poor health-seeking behavior and access to care as possible factors contributing to the deaths in the community. It is key to highlight the importance of providing regular community education on cholera signs and symptoms and prevention and treatment measures even in periods outside epidemics [12, 20, 21]. Capacity of community health volunteers should be built to educate community members on making water safe for drinking, regular hand washing with soap and other proper hygiene and sanitation practices, promote early reporting to health facilities and to report insanitary conditions that may predispose the community to future outbreaks to relevant authorities for action [18]. The relevant authorities should regularly inspect sanitation facilities to ensure good working order and to prevent and address problems arising from improper disposal of excreta and contamination of drinking sources.

While Akatsi did not have an epidemic preparedness response plan, there were a number of documentations on activities including end of outbreak report. KEEA on the other hand had a preparedness plan that guided response activities and ultimately, the attack rate was relatively lower. Reports on the outbreak activities and response were however very limited. Preparedness plans and documentation on outbreak response activities and post outbreak assessments are key documents in outbreak prevention and control [12]. The plan outlines important preparatory elements to be put in place and activities to implement including coordinating structures, logistics and training to ensure an effective response is mounted in the event of an outbreak. Given that risk factors for cholera outbreaks exist in Ghana, it would be expedient for all districts to have cholera preparedness plans that can be updated as necessary and activated when the need arises. Documenting events of outbreaks including compiling complete epidemiological surveillance data, analyses on time, person and place, response activities and the retrospective evaluation of the outbreak identifies strengths and weakness and pertinent information which can be used to improve planning for future epidemics.

The data for this paper was obtained from available documents as well as self-reports from the District Health Management Team staff associated with inherent issues of recall bias, limited avenues for independent verification and missing data. Despite these limitations, the assessment provides insightful information on the assessment of response activities carried out following cholera outbreaks, an area with limited published data especially from countries like Ghana which is endemic for cholera. Along the hierarchy from district to national levels, various stakeholders responsible for epidemic preparedness and response may learn from the experiences documented to inform and enhance policies and measures for cholera prevention and control activities in their relevant jurisdictions.

## Conclusion

In conclusion, this paper on the events and response activities in 2 cholera outbreaks in KEEA and Akatsi Districts has thrown light on strengths and areas that were overlooked or needed improvement for a better response. While in the long term resources are invested to improve potable water sources, sanitation and waste disposal, lessons can be learnt that can inform short term effective prevention and control measures which can be instituted to limit negative outcomes of cholera outbreaks in the country.

## Additional file

Additional file 1: Multilingual abstracts in the six official working languages of the United Nations. (PDF 639 kb)

## Abbreviations

DHMT: District Health Management Team
KEEA: Komenda-Edina-Eguafo-Abirem
WHO: World Health Organization
