# Supplementary material for: Assessment of the response to cholera outbreaks in two districts in Ghana
Source: Infect Dis Poverty. 2016 Nov 2;5:99. doi: 10.1186/s40249-016-0192-z (PMC5090876; doi:10.1186/s40249-016-0192-z)
Supplement: Additional file 1: — Multilingual abstracts in the six official working languages of the United Nations. (PDF 639 kb) [file 40249_2016_192_MOESM1_ESM.pdf]

Translation of the abstract into the six official working languages of the United Nations

تقييم الاستجابة لتفشي الكوليرا في مقاطعتين في غانا

سالي-آن اوهين، ويزدوم كلينيوي، مارك ساربي

خلاصة

خلفية: على الرغم من التفشي المتكرر للكوليرا في غانا، فقد قدمت تقارير قليلة عن تقييمات فعاليات الاستجابة للتفشي المتخذة في المناطق المتأثرة. قيّمت هذه الدراسة فعاليات الاستجابة المتخذة في مقاطعتين، مقاطعة (اكاتسي) في منطقة (فولتا) و بلدية (كوميندا-ايدينا-ايغوافو-ابيرم) (KEEA) في المنطقة الوسطى خلال وباء كوليرا عام 2012 في غانا.

الطرائق: أجرينا تقييماً استعادياً للأحداث، ونقاط القوة والضعف لفعاليات الاستجابة لتفشي الكوليرا في المقاطعتين باستخدام أداة تقييم الكوليرا الخاصة بالمنظمة العالمية للصحة. تضمنّت مصادر المعلومات المراقبةَ وتسجيلات المرافِق، وتقارير ومقابلات مع موظفي الصحة ذوي العلاقة بالاستجابة للتفشي من مرافق ومديريات الصحة الخاصة بالمقاطعتين. جمعنا البيانات المتعلقة بالعمر، والجنس، ومحل السكن، وتاريخ الابلاغ عن حالات الكوليرا للمؤسسات الصحية، وتعداد السكان في المقاطعة ومعلومات عن فعاليات الاستجابة للتفشي وأجرينا تحليلا استعادياً لبيانات التفشي بالنسبة للشخص، الوقت، والمكان.

النتائج: كان تفشي الكوليرا في (أكتاسي) متفجراً بمعدّل هجمات (AR) عال بلغ 100000\374 ومعدّل اماتة الحالات (CFR) بلغ 1.2% فيما كان ذلك في مقاطعة (KEEA) بمستوى أقل نسبياً بمعدّل هجمات 100000\23 ولكن بمعدّل اماتة حالات أعلى 18.8%. وفي كل من المقاطعتين، حددنا نقاط قوة متعددة في الاستجابة للتفشي من ضمنها ابلاغ مسؤولي الصحة في المقاطعة بالوقت المناسب والذي أطلق تحريا عاجلا للتفشي المحتمل ممهداً لتأكيد الكوليرا وابتداء فعاليات الاستجابة للصحة العامة. كانت الأخرى تنسيق الفعاليات المجراة بواسطة لجان متعددة القطاعات، وارساء مقاييس تعقيم ونظافة المياه وادارة الحالات الملائمة في المرافق الصحية. كما و وجدنا مواطن احتاجت للتحسين في كل من المقاطعتين بضمنها بيانات المراقبة غير المكتملة، المراقبة المجتمعية أقل من المناسبة باعتبار الابلاغ المتأخر والوفيات في المجتمعات ومعرفة المجتمع الشحيحة حول الاجراءات الوقائية للكوليرا.

الاستنتاجات: أشّر تقييم الاستجابة لتفشي الكوليرا في المقاطعتين نقاط قوة في فعاليات السيطرة على الأوبئة. ولكن كان هنالك حاجة لتعزيز الاستعداد وخصوصا في مجال تحسين المراقبة المجتمعية والوعي بالوقاية من الكوليرا وأهمية طلب العلاج العاجل من المرافق الصحية في حال حدوث تفشٍ للمرض.

Translated from English version into Arabic by Muther J. Alohmayed, through

TRANSLATORS WITHOUT BORDERS

加纳两区霍乱暴发的应急反应评估

Sally-Ann Ohene, Wisdom Klenyuie, Mark Sarpeh

摘要：

引言：尽管加纳再次暴发霍乱，但是几乎没有关于受影响地区暴发应急处置评估的报道。本研究评估 2012 年加纳两个区（Volta 地区的 Akatsi 区和中部地区的 Komenda-Edina-Eguafo-Abirem (KEEA)市）霍乱暴发的应急处置情况。

方法：通过 WHO 霍乱评价工具对以上两区的霍乱暴发应急处置情况、优势和不足进行分析。信息来源包括监测和机构记录、两区涉及暴发应急处置的卫生指挥部门和卫生机构的有关医务人员的采访。收集的数据包括年龄、性别、住址、霍乱病例报告时间、区人口信息和霍乱应急处置信息，并进行人群、时间和空间的描述性分析。

结果：Akatsi 区的霍乱呈暴发性，罹患率和病死率分别达 374/100 000 和 1.2%。KEEA 市规模相对较小，罹患率为 23/100 000，但病死率高达 18.8%。两区均表现出暴发应急处置的众多优势，包括及时向区卫生官员报告，区卫生官员及时对疑似暴发开展调查、从而促进暴发的核实和公共卫生应急处置的启动。其他还包括多部门协调，建立水、厕所并采取卫生设施，在医疗机构对病例进行适当管理等。两区也均存在需要改进的地方，如监测数据不完整、社区监测不理想（鉴于社区报告的延迟和死亡而言）以及社区对霍乱预防措施方面知识的不足。

结论：两区霍乱暴发处置的评估表明它们在暴发控制方面都有一些亮点，但是需要加强一些前期准备工作，尤其是改善社区监测、提高社区对霍乱预防及在霍乱发生时及时到卫生机构寻求治疗的认识。

Translated from English version into Chinese by Men-Bao Qian

CHINESE CENTER FOR DISEASE CONTROL AND PREVENTION
NATIONAL INSTITUTE OF PARASITIC DISEASES

Évaluation de la riposte aux épidémies de choléra dans deux districts du Ghana

Sally-Ann Ohene, Wisdom Klenyuie, Mark Sarpeh

Résumé

Contexte: malgré la récurrence des épidémies de choléra au Ghana, seuls de très maigres informations relatives aux évaluations des activités de lutte contre l'épidémie entreprises dans les régions affectées ont été communiquées. La présente étude a permis d'évaluer les activités de lutte entreprises dans deux districts, celui d'Akatsi dans la région de la Volta et le district municipal de Komenda-Edina-Eguafo-Abirem (KEEA) dans la région Centre, au cours de l'épidémie de choléra de 2012 au Ghana.

Méthodes: nous avons mené une évaluation rétrospective des événements, des points forts et des faiblesses des activités de lutte contre l'épidémie de choléra dans les deux districts en ayant recours à l'outil d'évaluation du choléra de l'OMS. Nos sources d'informations comprenaient les registres de surveillance et de différentes installations, les rapports et les entretiens réalisés avec le personnel de santé participant à la lutte contre l'épidémie et provenant à la fois des directions de la santé des districts et d'établissements de soins de santé. Nous avons collecté des données sur l'âge, le sexe, la zone de résidence, la date de signalisation de cas de choléra à l'établissement de soins de santé, les données de population du district et les activités de lutte contre l'épidémie avant de réaliser des analyses descriptives des données de l'épidémie en fonction des personnes, du temps et du lieu.

Résultats: l'épidémie de choléra à Akatsi a été explosive et s'est caractérisée par un taux d'attaque élevé (AR) de 374/100 000 et un taux de létalité (CFR) de 1,2 %. Cette épidémie a eu lieu à une échelle relativement plus réduite à KEEA, l'AR atteignant 23/100 000, mais le taux de létalité s'élevait lui à 18,8 %. Nous avons identifié des points forts dans la lutte contre l'épidémie dans les deux districts, dont l'avertissement rapide des responsables de santé du district, ce qui a permis de lancer un examen rapide de l'épidémie suspectée, facilitant ainsi la confirmation du choléra et le lancement d'activités de lutte de santé publique. Nous soulignons aussi l'utilité de la coordination des activités par des comités multisectoriels à l'origine de mesures d'approvisionnement en eau, d'assainissement et d'hygiène ainsi que de la gestion appropriée des cas au sein des établissements de soins de santé. Nous avons néanmoins également identifié certains aspects nécessitant des améliorations dans les deux districts, dont la présence de données de surveillance incomplète, la surveillance communautaire sous-optimal au regard de la signalisation tardive et des décès constatés au sein de la communauté et les connaissances inadéquates de la communauté à propos des mesures de prévention du choléra.

Conclusions: l'évaluation de la lutte contre l'épidémie de choléra dans les deux districts a souligné les points forts des activités de lutte contre l'épidémie. Il s'est néanmoins avéré nécessaire de renforcer la préparation, notamment en termes d'amélioration de la surveillance communautaire et de la sensibilisation à la prévention du choléra ainsi que de la nécessité de consultation rapide auprès d'établissements de soins de santé en cas d'épidémie.

Translated from English version into French by eric ragu, through

TRANSLATORS
WITHOUT BORDERS

Оценка мер реагирования на вспышку холеры в двух районах Ганы

Салли-Анн Оэнн, Висдом Кленьюи, Марк Сарпех

Резюме

Краткая информация: Несмотря на многократные вспышки холеры в Гане, мало исследований, анализирующих эффективность мер по реагированию на пострадавших от эпидемий территориях. Данная

работа анализирует меры по реагированию в двух районах – в Акатси в регионе Вольта и в муниципалитете Коменда-Эдина-Эгуафо-Абирем (КЭЭА) в Центральном районе во время эпидемии холеры в Гане в 2012 г.
Методы: Нами выполнена ретроспективная оценка событий, преимуществ и недостатков предпринятых мер по реагированию в вышеназванных районах на основании критериев ВОЗ. В качестве источников информации были записи контрольных органов и лечебных учреждений, отчеты и опросы задействованного в ликвидации эпидемии персонала административных и лечебных учреждений двух районов. Нами проанализирована информация о возрасте, поле, месте жительства, времени обращения за медицинской помощью, населении регионов и информация об ответных мерах на вспышку эпидемии, а также выполнен описательный анализ на основе персональных данных, данных о времени и месте.
Результаты: Вспышка холеры в Акатси отличалась стремительностью, высоким уровнем интенсивности атаки 374/100 000 и процентом смертности равным 1,2 %, в то время как в КЭЭА уровень интенсивности атаки был ниже - 23/100 000, но процент смертности выше – 18,8%. Нами выявлено достаточно сильных сторон органов здравоохранения обоих районов, вовремя оповестивших о подозрении на эпидемию, что позволило незамедлительно убедиться в наличии угрозы общественному здоровью и начать предпринимать меры по её предотвращению. Также, нами дана положительная оценка координационной деятельности межотраслевых комитетов, ответственных за водоснабжение, санитарный и гигиенический контроль, менеджмент здравоохранительных учреждений. Однако, на территории обоих районов выявлены недостатки, в том числе неэффективный общественный контроль, несвоевременные обращения за помощью и извещения о смертельных случаях, недостаточная информированность о мерах по предотвращению.
Выводы: Оценка реакции на вспышку холеры в двух районах показала результативность эпидемиологического контроля. Вместе с тем, выявлена необходимость, прежде всего, в повышении готовности к совершенствованию мер обществненого контроля и к повышению информированности о мерах по предотвращению и вариантов адекватного лечения в учреждениях здравоохранения во время эпидемии.

Translated from English version into Russian by Ms Zhdanova, through

TRANSLATORS
WITHOUT BORDERS

Evaluación de la respuesta a brotes de cólera en dos distritos en Ghana

Sally-Ann Ohene, Wisdom Klenyuie, Mark Sarpeh

Resumen
Antecedentes: a pesar de los recurrentes brotes de cólera que se han producido en Ghana, existen muy pocos informes de evaluación de las actividades de respuesta ante los brotes que se hayan aplicado en las áreas afectadas. El presente estudio evaluó las actividades de respuesta realizadas en dos distritos, Akatsi en la región del Volta y Komenda-Edina-Eguafo-Abirem (KEEA) en la región Central, durante la epidemia de cólera del año 2012 en Ghana.
Métodos: realizamos una evaluación retrospectiva de los acontecimientos y los puntos fuertes y débiles de las actividades de respuesta ante el brote de cólera en dos distritos mediante la herramienta de evaluación del cólera de la OMS. La información se obtuvo de registros de vigilancia e instalaciones, informes y entrevistas con personal sanitario relevante involucrado en la respuesta ante el brote, tanto de las direcciones sanitarias de los distritos como de las instalaciones sanitarias. Recogimos datos sobre edad, sexo, zona de residencia, fecha de notificación de los casos de cólera a las instalaciones sanitarias y datos de población del distrito, así como información sobre las actividades de respuesta ante el brote y los análisis descriptivos realizados sobre datos del brote desglosados por persona, tiempo y lugar.
Resultados: el brote de cólera en Akatsi fue explosivo, con una tasa de ataque (TA) de 374/100.000 y una tasa de letalidad (TL) del 1,2%, mientras que el de KEEA tuvo una TA algo menor, de 23/100.000, pero una alta TL del 18,8%. En ambos distritos se identificaron numerosos puntos fuertes en la respuesta al brote, incluyendo la notificación a tiempo a los funcionarios sanitarios del distrito, que dio lugar a una rápida investigación de la sospecha de brote, facilitó la confirmación del cólera y el inicio de las actividades de respuesta de sanidad pública. Además, se coordinaron las actividades desde comités multisectoriales, que implementaron medidas de agua, saneamiento e higiene y una gestión adecuada de los casos en las instalaciones sanitarias. También detectamos áreas

en ambos distritos en las que se necesitaban mejoras, tales como la insuficiencia de datos de vigilancia, un nivel de vigilancia basada en la comunidad inferior al óptimo teniendo en cuenta el retraso en la presentación de informes y las muertes en la comunidad y el inadecuado conocimiento de medidas de prevención del cólera por parte de la comunidad.
Conclusiones: la evaluación de la respuesta ante el brote de cólera en los dos distritos mostró las fortalezas de las actividades de control de epidemias. Sin embargo, se reveló la necesidad de reforzar la preparación especialmente en el campo de la mejora de la vigilancia y la sensibilización de la comunidad sobre la prevención del cólera y la importancia de buscar tratamiento temprano en instalaciones sanitarias en caso de que se produzca un brote.

Translated from English version into Spanish by Denis Smyth, through

TRANSLATORS
WITHOUT BORDERS
